# Personal preferences and discordant prostate cancer treatment choice in an intervention trial of men newly diagnosed with localized prostate cancer

**DOI:** 10.1186/1477-7525-10-123

**Published:** 2012-09-28

**Authors:** Jaclyn LF Bosco, Barbara Halpenny, Donna L Berry

**Affiliations:** 1 Dana-Farber Cancer Institute, 450 Brookline Avenue, LW 519, Boston, MA, 02215, USA

**Keywords:** Localized prostate cancer, Randomized trial, Decision making, Treatment, Patient preferences, Quality of life

## Abstract

**Background:**

Men diagnosed with localized prostate cancer (LPC) can choose from multiple treatment regimens and are faced with a decision in which medical factors and personal preferences are important. The Personal Patient Profile-Prostate (P3P) is a computerized decision aid for men with LPC that focuses on personal preferences. We determined whether the P3P intervention improved the concordance of treatment choice with self-reported influential side-effects compared with a control group.

**Methods:**

English/Spanish-speaking men diagnosed with LPC (2007–2009) from four US cities were enrolled into a randomized trial and followed through 6-months via mailed or online questionnaire. Men were randomized to receive the P3P intervention or standard education plus links to reputable websites. We classified choice as concordant if men were concerned with (a) sexual function and chose external beam radiotherapy or brachytherapy, (b) bowel function and chose prostatectomy, (c) sex, bowel, and/or bladder function and chose active surveillance, or (d) not concerned with any side effect and chose any treatment. Using logistic regression, we calculated odds ratios (OR) and 95% confidence intervals (CI) for the association between the P3P intervention and concordance.

**Results:**

Of 448 men, most were <65 years, non-Hispanic white, had multiple physician consultations prior to enrollment, and chose a treatment discordant with concerns about potential side effects. There was no significant difference in concordance between the intervention (45%) and control (50%) group (OR = 0.82; 95%CI = 0.56, 1.2).

**Conclusions:**

The P3P intervention did not improve concordance between potential side effects and treatment choice. Information and/or physician consultation immediately after diagnosis was likely to influence decisions despite concerns about side effects. The intervention may be more effective before the first treatment options consultation.

**Trial registration:**

NCT00692653 http://clinicaltrials.gov/ct2/show/NCT00692653

## Introduction

Over 218,000 men are diagnosed with prostate cancer each year in the United States (US), of whom 90% are classified as having clinically localized prostate cancer (LPC) [1]. Men diagnosed with LPC choose from multiple treatment or surveillance regimens with the goal to increase survival and minimize treatment side effects [2,3]. Treatment options include radical prostatectomy, external beam radiotherapy, brachytherapy, cryosurgery, high intensity focused ultrasound (HIFU), and active surveillance (no active treatment, but PSA levels are monitored) [4]. In a prospective study of men diagnosed with LPC, sexual dysfunction (50%) was the most common side effect one year after radical prostatectomy, while bowel dysfunction (9%) was the most common side effect after external beam radiotherapy and brachytherapy [5]. The lack of evidence to show that one of these active treatment options results in a better survival advantage than others, except when men have higher risk disease [6], adds to the complexity of treatment decision-making for men with LPC [7-9]. Therefore, men are faced with a decision that may incorporate personal preferences based on potential side effects from certain treatments, their current health history, and lifestyle and family concerns [10].

The Decision Support Framework (DSF) [11] is organized by the 1) determinants of decisions, 2) decision support interventions, and 3) evaluation of both the process and outcomes of the decision support. While the DSF is based on expectancy value, decisional conflict and social support theories, it also addresses complex situations faced by patients in contemporary health dilemmas [11]. Deciding about treatment of LPC is one of the most appropriate decisions that fit within this framework because it encompasses careful deliberation of demographic, clinical, practice environment, perceptions of both the decision and of importance of others involved in the decision, and resources to make and implement the decision.

The Personal Patient Profile-Prostate (P3P) is a computerized, decision support system, developed under the conceptual approach to healthcare decision making using the DSF, for men with LPC that focuses on personal preferences [12]. Using data from the P3P trial, we determined whether the P3P intervention improved the concordance of treatment choice with self-reported influential side-effects compared with a control group.

## Methods

### Study population

A detailed description of methods from the P3P interventional trial has been reported elsewhere [12]. Briefly, men diagnosed with LPC were enrolled into a randomized trial between March 2007 and November 2009 that evaluated a computerized prostate cancer intervention used to aid in LPC treatment decision making. Men were enrolled from four geographically diverse areas in the United States. Specifically, men were enrolled from the Seattle Prostate Institute, University of Washington Seattle Cancer Care Alliance, or the Puget Sound Veteran’s Affairs Hospital in Seattle, Washington, the Veteran’s Affairs Hospital in San Antonio, Texas, Fox Chase Cancer Center in Philadelphia, Pennsylvania or the Veteran’s Affairs Hospital in Augusta, Georgia. At the three Veteran’s Affairs hospitals, men had access to all treatment options regardless of ability to pay. At all enrollment sites, eligible men were determined by their physician to be potential candidates for all treatment options. The Institutional Review Boards of the study coordinating center, the Fred Hutchinson Cancer Research Center/University of Washington Cancer Consortium, and all of enrolling sites, approved the study, and participants provided written consent.

Men diagnosed with histologically confirmed T1 or T2 prostate cancer of any risk level, which could speak and read English or Spanish at a 6^th^ grade level or higher, and were consulting with cancer specialists about their treatment options but had not started therapy, were eligible for the trial. All consenting participants (N = 494) completed the Web-based Personal Patient Profile – Prostate (P3P) query component at baseline either on touch-screen computers in the clinic waiting rooms prior to the consult visit with a cancer specialist, or at home for men who had broadband Internet access. Privacy was maintained by the use of private consultation rooms or corners of waiting rooms where only the participant could view and hear the computer through privacy screens and headphones.

The P3P query component was administered at enrollment (baseline) and included measures of demographic characteristics, influential personal factors, information priorities, and current symptoms [13]. Participants were followed at 1-month and 6-months after enrollment through mailed or online questionnaires. For this analysis we evaluated the concordance between concerns about bladder function, sexual function and/or bowel function as potential treatment side effects reported at enrollment, and 6-month treatment choice; therefore men who did not return the 6-month follow-up questionnaire were not eligible for this analysis (n = 60).

### Analytic variables

Randomization to the intervention or control group is described in detail by Berry *et al.*[12]. After completing the P3P query component at enrollment, participants were allocated automatically by the program to the P3P intervention software program (intervention), a computerized decision support system providing customized text and video coaching regarding the relationship between treatments and potential side effects, influential personal factors, and communication with health care providers [13], or standard of care plus links to reputable websites that provided basic information about prostate cancer (control). Each participant in the intervention group received brief on-screen narratives/videos plus printable teaching sheets on five topics (prognosis, stage, treatment options, side effects, sexuality) and the option to receive one or more teaching sheets on home care in terms of the impact on family and family risk of prostate cancer.

In the P3P query component, all men (intervention and control group) self-reported one of four Likert-type options for the importance or influence of potential side effects on their treatment decision (“a lot of influence”, “some influence”, “a little influence”, or “no influence”): (a) bladder dysfunction, (b) sexual dysfunction, and/or (c) bowel dysfunction. Responses were dichotomized as “a lot” versus “some, a little and no” influence. Participants were queried about influential personal factors before indicating priority information needs and before receiving any of the customized information and coaching.

Self-reported prostate cancer treatment choice was collected at 1- and 6-months after study enrollment. Men reported choosing active surveillance, external beam radiotherapy, brachytherapy, cryosurgery, prostatectomy, HIFU, or other treatments. Participants in this trial were specifically asked about “watchful waiting”, but the contemporary treatment approach of no active treatment, but monitored PSA levels is “active surveillance”; therefore, we refer to this treatment approach as “active surveillance”. Those who returned the questionnaires but did not report a final treatment decision by the 6-month follow-up questionnaire were classified as active surveillance. Among men who returned the 6-month follow-up questionnaire, but did not specify a treatment choice (n = 31), we carried forward any treatment choice previously reported at 1-month.

Treatment choices were classified as concordant when men reported that (a) sexual function influenced their decision and they ultimately decided by 6-months to have external beam radiotherapy or brachytherapy, (b) bowel function influenced their decision and they chose radical prostatectomy, c) sexual, bowel, and/or bladder function influenced their decision and they chose active surveillance, or d) none of the concerns influenced their decision and they chose any treatment approach. Because bladder dysfunction is a side effect of both prostatectomy and radiotherapy, participants concerned with bladder function were discordant for prostatectomy as well as external beam radiotherapy and brachytherapy, but concordant with active surveillance.

### Potential confounding variables

Information on socio demographic and clinical characteristics were collected at enrollment via the P3P query component and included age, weeks since biopsy, prior doctor’s visit, full-time work status, college education, married/partnered, annual household income, minority race/ethnicity, and health insurance. State and trait anxiety were measured at enrollment with the Spielberger State-Trait Anxiety Inventory [14], and self-reported Expanded Prostate Index Composite-Short Form (EPIC-26) [15] was collected at enrollment and 6-months.

At enrollment, men also reported the influence that length of expected survival, other people, age, lifestyle activities, and confidence in a particular consulting physician had on their treatment choice. For each *influential person* (co-workers/colleagues, other friends, spouse/partner, other family members, and famous individuals with a diagnosis of prostate cancer), we assigned a score of 0 to 3 for men who reported that their treatment decision was influenced “none”, “a little”, “some”, and “a lot”, respectively. The scores were summed across all *influential people* to calculate an overall “influential people score”. A similar calculation was made across *recreation/leisure activities* and *work/occupational activities*. The responses for *length of survival* and *confidence or lack ofconfidence in a particulardoctor* were also assigned scores of 0 to 3 for “none”, “a little”, “some”, and “a lot”. Men were also asked to report if age influenced their treatment decision (yes, no). No information on Gleason score, PSA levels, D’Amico risk score, or comorbidities was collected.

### Statistical analysis

We described socio demographic and clinical characteristics, influential people and factors, and EPIC symptoms using frequencies and proportions for categorical variables, and means and standard deviations for continuous variables. We calculated the frequency and proportion of men whose combination of influential side effects were concordant with their treatment choice. Chi-square tests were used to determine if the proportion of concordance was significantly different between the intervention and control groups. After preliminary analysis, cryotherapy (n = 3), HIFU (n = 2), and other (hormonal, naturopathic, or alternative medicine) treatments (n = 7) were excluded from further analyses because too few men chose these treatments.

Odds ratios (OR) and 95% confidence intervals (CI) were calculated for the association between study group and concordance using logistic regression. Confounding variables were identified *a priori* from our previous work (study site, age at enrollment, current work status, health insurance, Internet as an information source, trait anxiety, and the influence of people, age, recreational or work activities, confidence or lack of confidence in a particular doctor, and expected survival) [12]. We repeated the analysis stratified by treatment choice to assess whether the results differed by specific treatments. All analyses were conducted using SAS version 9.2 (Cary, North Carolina).

## Results

After excluding 46 men due to reasons stated above, the total analytic sample included 448 men. Excluded participants did not differ by socio demographic characteristics (data not shown). The majority of the sample was non-Hispanic white, currently married/partnered, received Medicare, State, or Private health insurance, used the Internet as a prostate cancer information source, and saw at least one physician prior to enrollment (Table 1). Radical prostatectomy was the most common treatment choice by 6-months (50%).

**Table 1:** **Descriptive characteristics, EPIC symptoms,and personal influences ina randomized controlled trialof men with localizedprostate cancer (N =** **448)**

**Characteristic**	**Overall**		**Concordant**		**Not Concordant**	
	**N = 448**		**N = 211**		**N = 237**	
	**N**	**%**	**N**	**%**	**N**	**%**
Intervention group	239	53	107	51	132	56
Study site						
Augusta, GA - Veteran’s Affairs	81	18	43	20	38	16
Philadelphia, PA - Fox Chase Cancer Center	79	18	26	12	53	22
San Antonio, TX - Veteran’s Affairs	21	4.7	14	6.6	7	3.0
Seattle, WA - Seattle Prostate Institute	18	4.0	10	4.7	8	3.4
University of Washington – Seattle Cancer Care Alliance	207	46	96	46	111	47
Seattle, WA - Puget Sound Veteran’s Affairs	42	9.4	22	10	20	8.4
Age 65 or older at enrollment	175	39	89	42	86	36
4 or more weeks since biopsy	297	66	141	67	156	66
Prior doctor's visit	318	71	139	66	179	76
Minority race/ethnicity	71	16	42	20	29	12
Currently married or partnered	352	79	168	80	184	78
College degree or higher	255	57	116	55	139	59
Full-time work status	206	46	90	43	116	49
<$35,000 annual household income	100	22	57	27	43	18
Health Insurance						
None or missing	16	3.6	6	2.8	10	4.2
VA or Military	96	21	55	26	41	17
Medicare, State, or Private	336	75	150	71	186	78
Internet as an information source	314	70	135	64	179	76
Influenced by age	276	62	120	57	156	66
Treatment choice by 6-months						
Active surveillance	68	15	39	18	29	12
External Beam Radiotherapy	94	21	28	13	66	28
Brachytherapy	63	14	23	11	40	17
Radical Prostatectomy	223	50	121	57	102	43
	**Mean ±**	**St Dev**	**Mean ±**	**St Dev**	**Mean ±**	**St Dev**
State anxiety	40.2 ±	13.2	38.1 ±	12.5	42.0 ±	13.5
Trait anxiety	33.4 ±	10.2	33.1 ±	9.6	33.6 ±	10.7
Baseline EPIC hormonal	88.7 ±	12.8	89.1 ±	13.1	88.4 ±	12.6
Baseline EPIC urinary irritative	84.4 ±	15.1	84.5 ±	13.6	84.2 ±	16.4
Baseline EPIC urinary incontinence	92.9 ±	13.3	91.6 ±	14.4	93.9 ±	12.2
Baseline EPIC bowel	94.4 ±	10.2	94.6 ±	10.5	94.2 ±	9.8
Baseline EPIC sexual	64.1 ±	29.8	60.2 ±	31.2	67.5 ±	28.3
6-month EPIC hormonal	85.4 ±	16.6	87.0 ±	15.9	84.0 ±	17.2
6-month EPIC urinary irritative	82.6 ±	17.8	83.3 ±	17.4	82.0 ±	18.2
6-month EPIC urinary incontinence	73.3 ±	29.3	69.4 ±	30.4	76.8 ±	27.8
6-month EPIC bowel	90.3 ±	14.5	90.5 ±	14.5	90.1 ±	14.5
6-month EPIC sexual	40.1 ±	31.9	35.1 ±	30.4	44.6 ±	32.5
Index for influential people	6.1 ±	3.0	5.7 ±	3.0	6.5 ±	2.9
Index of recreational and work activities	3.9 ±	1.8	3.4 ±	1.9	4.3 ±	1.6
Influenced by expected survival	2.6 ±	0.81	2.4 ±	0.93	2.7 ±	0.65
Influenced by confidence or lack of confidence in a particular doctor	2.5 ±	0.90	2.3 ±	1.0	2.7 ±	0.69

All three concerns (bladder, sexual and bowel function) was the most commonly reported combination of side effects that influenced treatment decisions in our sample (33%), followed by bowel function (27%), and then no influence of any of the three potential concerns (24%). Overall, 47% chose a treatment concordant with their concerns about influential side effects by 6-months after enrollment. There were no significant differences in the proportion of concordance between the intervention (45%) and control (50%) group overall (p = 0. 29) or by specific treatment choice (Table 2). Among men who chose active surveillance (n = 68), concordance was 45% in the intervention and 58% in the control group (p = 0.05), while concordance for men who chose brachytherapy (n = 63) was 36% in the intervention and 37% in the control group (p = 0.97). Treatment concordance among men who chose external beam radiotherapy (n = 94) was 32% in the intervention and 28% in the control group (p = 0.65), and for those who chose prostatectomy (n = 223), 53% in the intervention and 56% in the control group (p = 0.71).

**Table 2:** **Frequency and proportion ofthe 211 men inthe P3P intervention andcontrol group who madea concordant treatment choiceby 6-months given theirconcerns about influential sideeffects (bladder, sex, andbowel) at enrollment**

**Influential Side Effects**	**Overall**		**Active surveillance**		**External Beam Radiotherapy**		**Brachytherapy**		**Radical Prostatectomy**	
	**Intervention N = 107**	**Control N = 104**	**Intervention N = 15**	**Control N = 24**	**Intervention N = 15**	**Control N = 13**	**Intervention N = 16**	**Control N = 7**	**Intervention N = 61**	**Control N = 60**
	**N (%)**	**N (%)**	**N (%)**	**N (%)**	**N (%)**	**N (%)**	**N (%)**	**N (%)**	**N (%)**	**N (%)**
All ^**a**^	8 (7.5)	14 (13)	8 (53)	14 (58)	0 (0)	0 (0)	0 (0)	0 (0)	0 (0)	0 (0)
Bowel ^**b**^	31 (29)	32 (31)	0 (0)	0 (0)	0 (0)	0 (0)	0 (0)	0 (0)	31 (51)	32 (53)
Sex ^**c**^	7 (6.5)	8 (7.7)	0 (0)	0 (0)	5 (33)	7 (54)	2 (13)	1 (14)	0 (0)	0 (0)
Bowel and Sex ^**d**^	0 (0)	1 (1.0)	0 (0)	1 (4.2)	0 (0)	0 (0)	0 (0)	0 (0)	0 (0)	0 (0)
Bladder ^**e**^	1 (0.9)	2 (1.9)	1 (6.7)	2 (8.3)	0 (0)	0 (0)	0 (0)	0 (0)	0 (0)	0 (0)
None ^**f**^	60 (56)	47 (45)	6 (40)	7 (29)	10 (67)	6 (46)	14 (88)	6 (86)	30 (49)	28 (47)
p-value ^**g**^	0.29		0.05		0.65		0.97		0.71	

a. Concerned about bladder, sexual, and bowel dysfunction concordant with active surveillance.

b. Concerned about bowel dysfunction concordant with radical prostatectomy.

c. Concerned about sexual dysfunction concordant with external beam radiotherapy and brachytherapy.

d. Concerned about bowel and sex dysfunction concordant with active surveillance.

e. Concerned about bladder dysfunction concordant with active surveillance.

f. Not concerned about any influential side effects concordant with all treatment choices.

g. p-value to test difference in concordance between intervention and control group.

As seen in Table 3, the P3P intervention group, compared with the control group, was not associated with treatment concordance overall (OR = 0.82; 95%CI = 0.56, 1.2) or when restricted to those who chose external beam radiotherapy (OR = 1.2; 95%CI = 0.51, 3.0), prostatectomy (OR = 0.90; 95%CI = 0.53, 1.5). None of the confounding variables substantially altered our findings (Table 3). The results did not change when we excluded the 31 men who skipped the treatment choice question in the 6-month follow-up questionnaire. We evaluated whether varying the classification of the potential influence on treatment choice would alter our findings, but when we collapsed “a lot” and “some” influence with “a little” and “none”, the results between the intervention and concordance did not substantially change (data not shown).

**Table 3:** **Study group (intervention orcontrol group) in relationto the concordance betweeninfluential side effects and6-month treatment choice**

**6-month treatment choice**	**Cases**^**a**^**/ Total**^**b**^	**Unadjusted**		**Adjusted**^**c**^	
		**OR**	**95%CI**	**OR**	**95%CI**
All treatments	211 / 448	0.82	(0.56, 1.2)	0.76	(0.51, 1.2)
Active surveillance	39 / 68	0.38	(0.14, 1.0)	0.16	(0.03, 0.79)
External beam radiotherapy	28 / 94	1.2	(0.51, 3.0)	1.3	(0.49, 3.7)
Brachytherapy	23 / 63	0.98	(0.32, 3.0)	0.96	(0.11, 8.3)
Prostatectomy	121 / 223	0.90	(0.53, 1.5)	0.87	(0.49, 1.6)

a. Number of concordant cases.

b. Total number treated.

c. Adjusted for health care site, age at enrollment, full-time work status, health insurance type, internet as an information source for prostate cancer, trait anxiety, age as a factor in treatment choice, influence of recreational or work activities as considering treatment choice, importance or influence of confidence or lack of confidence in a particular doctor in treatment choice, and importance or influence of survival on treatment choice.

## Discussion

In a multi-center sample of men newly diagnosed with LPC, we observed that the majority of men who reported potential side effects of bladder, sexual, and/or bowel dysfunction as influential at enrollment, ultimately chose a treatment option within 6-months that contradicted their concerns about these side effects, regardless of receiving the P3P intervention or not.

Our results are consistent with previous studies [3,16-18]. As described by Zeliadt *et al*., a gap between men’s beliefs and knowledge regarding the efficacy and clinical evidence for prostate cancer treatment options has been identified, particularly among those who choose radical prostatectomy, which contributed to treatment choice mismatches in that study [3].

Men may minimize side effect concerns when they make a treatment decision. Side effects may be less important because men may not understand which side effect is an issue for a particular treatment option, information received is inaccurate, or the information was presented in a way that was too difficult to understand [3,7,16,19]. For example, Denberg *et al.* found that among 20 men diagnosed with LPC at a Veterans’ Administration Hospital, most confused radiation therapy with chemotherapy side effects, erroneously believing hair loss to be a common side effect of radiation therapy [16]. In a qualitative study of 18 men with prostate cancer, O’Rouke found that men perceived that radical prostatectomy and external beam radiotherapy resulted in the same side effects [19]. Additionally, in a study of knowledge and understanding of LPC treatment choices among 184 men [7], over 70% of patients incorrectly answered questions related to LPC treatment complications and prognosis in the absence of treatment, and these common misperceptions were prevalent even among those who had better socioeconomic and health profiles.

Our findings indicated a high proportion of men (71%) consulted with multiple physicians prior to enrollment. A majority of our sample may have relied on multiple consults to arrive at a treatment decision that synthesized recommendations received prior to study enrollment. This experience could explain the lack of impact for the P3P intervention on concordance. The best time to engage with a decision support system such as the P3P is likely sooner than later in the decision making process; thus implementing the intervention earlier, immediately after learning the biopsy results, could improve concordance as men are more able to consider and articulate each personal concern.

Contributing to the lack of concordance in our study was the fact that a third of our sample indicated that all three potential side effect concerns had “a lot of influence” on their treatment choice at enrollment. Participants responded to Likert-type scales to rate the importance of potential side effects. Because these men rated all of the potential side effects as equally important (tied scores) in our study, this produced a “ceiling-effect” and we could not discriminate the rank order. An alternative scaling method as described by Sloan *et al.*[20] uses Thurstone’s “law of comparative judgment” to reduce the number of ties, thus avoiding the “ceiling effect” by forcing respondents to choose each of the items over each other. However, in order to implement this method, 66 paired comparisons would have been necessary because the P3P query component includes 12 information priorities: three potential side effects, and nine other influences of people (co-workers/colleagues, other friends, spouse/partner, other family members, famous individual), activities (occupational or leisure), confidence in clinician, and survival; thus the paired comparisons method would have been overly burdensome to our participants.

Two observational studies by Barocas *et al.*[21] and Cooperberg *et al.*[22] utilized the Cancer of the Prostate Strategic Urologic Research Endeavor (CaPSURE) registry and reported that fewer than 10% of eligible men with LPC choose active surveillance among 1,886 between 1999 and 2004 and 11,892 participants when enrollment was extended through 2008, respectively. Among men who deemed all three potential side effects concerns important to their treatment choice in our study, only 8 men in the intervention group and 14 men in the control group chose active surveillance by 6-months. As reviewed by Pickles *et al.*[23], fewer men choose active surveillance because of increased anxiety from (a) the lack of intervening treatment, (b) uncertainty due to control loss, and (c) lack of education and support.

Radical prostatectomy was the most common treatment choice at 6-months in our study. At the time of the P3P trial, no LPC treatment approach had demonstrated any survival advantage over another. Regardless of educational level, Denberg *et al.*[16] found that men often have misconceptions about radical prostatectomy, including that removal of the tumor provides a substantial survival advantage over other treatment options. Denberg *et al.*[16] also reported that men also perceived that external beam radiotherapy was more convenient than surgery. Likewise, Diefenbach *et al*. [24] observed that men who chose radical prostatectomy perceived that their tumors were more serious than men who chose external beam radiotherapy.

We studied a relatively large cohort of men from four geographically diverse locations in the US. We also enrolled men from both urology and radiation oncology clinics, and used a prospective design to quantitatively capture the influence of potential side effect concerns prior to making a treatment decision. The P3P intervention trial was powered to detect a decrease in decisional conflict among men newly diagnosed with LPC [12], not to increase concordance between concern for side effects and treatment choice. All participants in the trial were confirmed to have a clinically diagnosed stage I or II localized prostate cancer, but information on Gleason score, PSA levels, D’Amico risk score, physician assessment of options, or comorbidities were not collected.

While we assessed the influence of socio demographic and clinical characteristics, in addition to influences of people, activities, age, and expected survival, our study may be biased by potential unknown decision-making influences or unmeasured tumor characteristics. Additionally, we were unable to abstract medical records from all participants to confirm treatment actually received. Finally, given the homogeneity of the sample, the generalizability of our findings may be limited.

## Conclusion

In conclusion, we observed that the majority of men with LPC made treatment decisions discordant with their concerns for potential side effects and the P3P intervention did not improve treatment concordance. Information and/or physician consultation immediately after diagnosis was likely to influence decisions despite concerns about side effects, as were other influential personal factors. The intervention may be more effective before the first treatment options consultation.

## Competing interest

There are no financial disclosures or conflicts of interest from any authors.

## Authors’ contributions

JLFB was responsible for the conception and design, data analysis and interpretation, and manuscript writing. DLB and BH were responsible for the conception and design, protocol development, patient recruitment, and manuscript writing. All authors read and approved the final manuscript.
